# Buccal window approach: A favorable technique for preserving periodontium in impacted third molar surgery

**DOI:** 10.34172/japid.025.3652

**Published:** 2025-08-04

**Authors:** Fatemeh Latifi, Azadeh Esmaeilnejad, Seyed Mohamad Mahdi Bahaodini, Shakila Peymani, Ardeshir Khorsand, Milad Baseri

**Affiliations:** ^1^Department of Oral and Maxillofacial Surgery, School of Dentistry, Shahid Beheshti University of Medical Sciences, Tehran, Iran; ^2^Department of Periodontics, School of Dentistry, Shahid Beheshti University of Medical Sciences, Tehran, Iran; ^3^Department of Oral and Maxillofacial Surgery, School of Dentistry, Isfahan University of Medical Sciences, Isfahan, Iran

**Keywords:** Buccal window, Impacted, Periodontium, Third molar

## Abstract

**Background.:**

The present study assessed the probing depth of the second molar distal aspect after the surgical removal of adjacent mandibular impacted third molars using osteotomy by a buccal window and routine crestal flaps.

**Methods.:**

In this double-blind, randomized clinical trial, 16 candidates for the surgical removal of bilateral mandibular third molars were selected, and each side’s tooth was assigned to a different osteotomy group. The surgery was performed using a sulcular flap and a distal extension for one group, while the osteotomy was performed using the buccal window technique. The pocket probing depth of the adjacent second molars was calculated before and three months after the surgical removal. The data of both groups were statistically analyzed using normality and t-tests in the SPSS software. Statistical significance was set at *P*<0.05.

**Results.:**

At three months postoperatively, significant differences were found between the two groups regarding pocket probing depth at mesiobuccal (5.23±1.12 mm in the crestal osteotomy and 4.03±1.16 mm in the buccal window osteotomy; *P*<0.006), mid-distal (5.77±1.08 mm in the crestal osteotomy and 4.25±1.35 mm in the buccal window osteotomy; *P*<0.002), and distolingual aspects (5.46±1.34 mm in the crestal osteotomy and 3.96±1.11 mm in the crestal osteotomy; *P*<0.002) of the adjacent second molars. The mean pocket probing depth of the mid-distal area was significantly lower in the buccal window osteotomy.

**Conclusion.:**

According to the results, this technique can be used as an alternative to crestal osteotomy in level C impactions and Cl I and Cl II impactions regarding position towards the anterior edges of the mandibular ramus.

## Introduction

 Mandibular third molars are the most frequently impacted teeth. The probable reasons for impaction include eruption age, space deficiency, and abnormal tooth bud position. Impaction or semi-eruption of the tooth could result in pericoronitis, resorption of the adjacent molar’s root, dental caries, and pathologic lesions.^1^ Therefore, the surgical removal of the third mandibular molar—whether prophylactic or therapeutic—is common.^2^

 Surgical extraction of mandibular third molars requires flap elevation and osteotomy. Postoperative tissue trauma and inflammation lead to postoperative complications such as pain, swelling, trismus, and ecchymosis. Prior investigations have predominantly assessed the influence of impacted mandibular third molar extraction on the periodontal status of adjacent dentition. Several studies have reported that early removal of impacted third molars may confer periodontal benefits, particularly distal to the second molars and within the corresponding sextant region.^3,4^ Conversely, other studies have indicated that such extractions may be associated with adverse periodontal outcomes, including defects in the distal root of the second molars, decreased alveolar bone height, increased attachment loss, and greater periodontal pocket depth in the distal aspect of the affected tooth.^5,6^ Several suggestions have been made, including avoiding extraction in complicated cases, different flap techniques, bone grafting, PRP or collagen, and changing the osteotomy technique.^7^

 Buccal window osteotomy was first introduced in 1999 to preserve the intact bone distal to the second mandibular molar and avoid vertical bone loss, subsequent pocket formation, dehiscence, and debris accumulation. Furthermore, this technique avoids the lingual flap required for the crestal osteotomy and its succeeding complications.^8^ This technique is suggested for a fully impacted third mandibular molar with sufficient bone at the coronal area.

 Most studies evaluating the periodontal status of the distal second mandibular molar have considered flap techniques, suturing, and the application of bone materials. The present study was designed to assess the distal pocket of the second mandibular molar after crestal osteotomy and buccal window.

## Methods

 This split-mouth study was a double-blind randomized clinical trial. This study was conducted in accordance with the guidelines outlined in the Declaration of Helsinki.^9^ After properly explaining the investigation’s aim and procedure, all the participants provided written informed consent forms.

 Considering the average expected clinical difference in two osteotomy techniques of 0.5 mm, and also including SD = 0.6 based on the results of Baqain et al’s review^7^ and taking into account the first type error of α = 0.05 and the second type error of β = 0.1, 26 subjects were required for each group in the research. However, due to technical limitations, we were only able to include 16 samples in each group during the study period.

 Sixteen healthy cases (ASA I-II), aged 18‒35, were enrolled in the study. All of them had two impacted third molars and were candidates for surgical extraction. The mandibular molars were assessed using a panoramic radiograph based on Pell & Gregory’s and Winter’s classifications. Only fully impacted teeth (Grade C), Class I and II, with vertical, mesial, and horizontal angulations were considered. The crestal bone height was ≥ 1 mm, and the distance between the impacted crown and the adjacent molar root was > 2 mm.

 The exclusion criteria included any systemic disease interfering with tissue healing, like diabetes mellitus, crowding, obvious malocclusion, poor oral hygiene, history of periodontal disease, and presence of pathological lesions. Pregnant or lactating women and smokers were omitted.

 One surgeon performed all surgical extractions. After a month, the surgery was performed on the other side using the other osteotomy technique. Due to this, both groups were similar, and the random allocation and differences between groups were eliminated. One dentist evaluated pocket depth before surgery and three months postoperatively. The researcher who analyzed the data differed from the surgeon and the dentist. The patient was not informed about the chosen osteotomy technique for each tooth.

**Figure 1:**
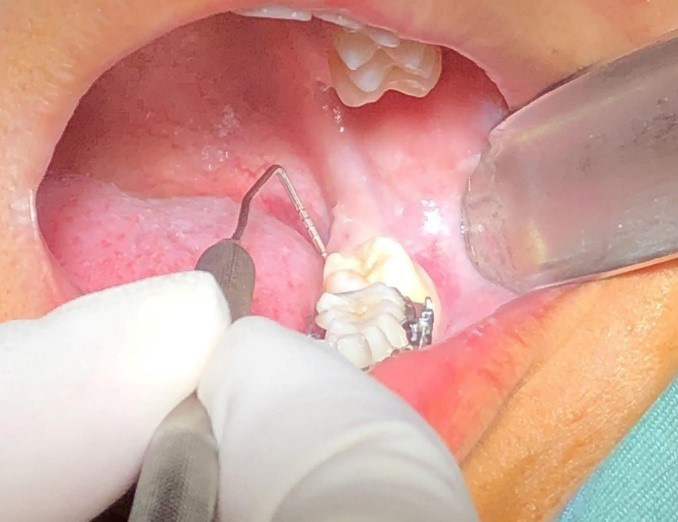
Measuring the probing depth of the distal pocket of the second mandibular molar before surgery using a Williams probe

**Figure 2:**
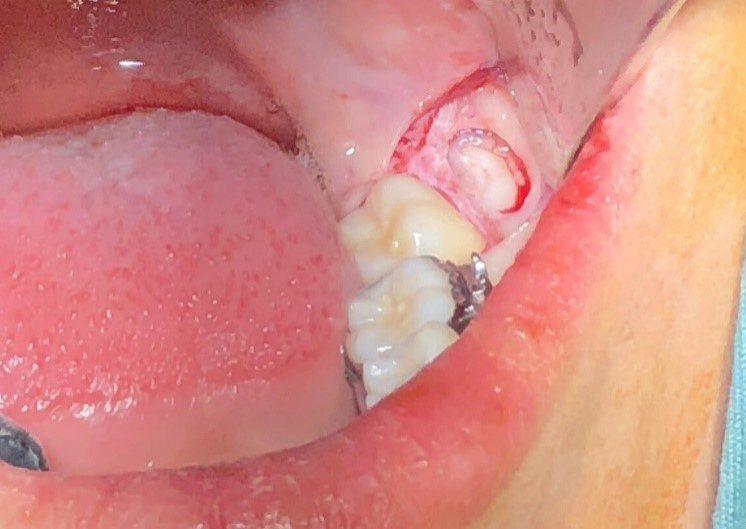
Buccal window osteotomy technique before extraction of the third mandibular molar

**Figure 3:**
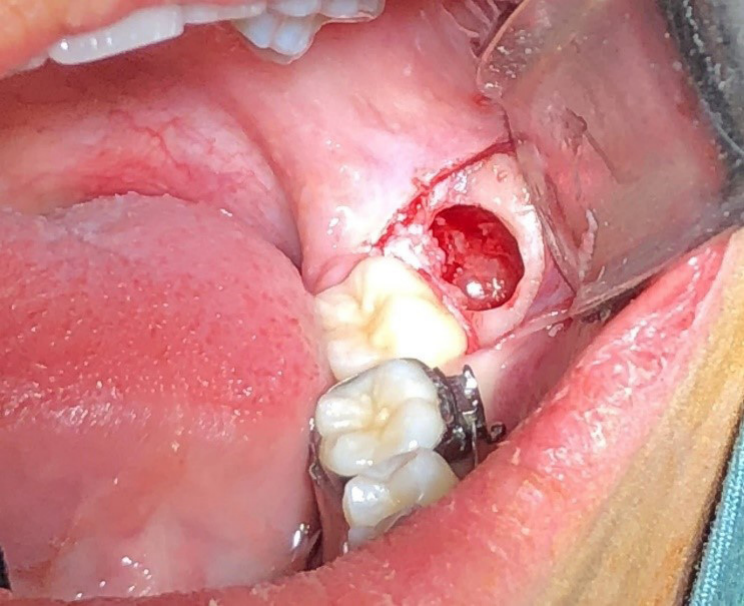
Buccal window osteotomy technique after the extraction of the third mandibular molar

 Before the surgery, three distal sites—distolingual, distobuccal, and mid-distal of the mandibular second molar—were examined. The probing depth was measured using a walking probing technique with a Williams probe (Figure 1). The Halsted block technique was used to achieve an inferior alveolar nerve block with 2% lidocaine containing 1:80,000 epinephrine. A sulcular incision was made at the second molar and extended distally. In the crestal osteotomy group, the approach to the impacted tooth involved bone removal from the crestal site. Tooth sectioning was performed if required. During the buccal osteotomy, the buccal bone covering the third mandibular molar was removed, preserving the crestal bone with a 2-mm distance from the crestal bone and a 2-mm distance from the root of the second mandibular molar. The window was formed until the whole crown of the mandibular third molar was visible (Figure 2). The tooth was then sectioned and removed using an elevator (Figure 3). The pocket depths were measured three months after the surgery.

 The depth, angulation, and relationship of the third molar tooth with the anterior edge of the ascending ramus were confounders of this study, which were slightly resolved by the fact that each patient underwent both types of osteotomies.

###  Statistical analysis

 Data were analyzed using SPSS 25 (IBM, Chicago, IL, USA). Clinical factors were reported using means and standard deviations, and t-tests were used to compare the two groups. The level of statistical significance was set at *P* < 0.05.

## Results

 Sixteen patients were enrolled in this study. The participants were 18‒25 years old (mean: 21 years), and 81% were female. Tables 1 and 2 present mean pocket depths and changes in each group before and three months after surgery.

 Three months after the operation, distal pockets of second mandibular molars at the crestal osteotomy site were significantly greater than those on the buccal osteotomy side.

 The buccal osteotomy group’s mid-distal pocket depth has been reduced by approximately 0.2 mm, while the crystal osteotomy showed a 1.4-mm increase.

**Table 1:** Mean distal pocket depth (mm) of the second mandibular molar in each group before and three months after surgery (T_0_: before surgery, T_1_: three months after surgery)

**Probing** **site**	**Time**	**Crestal osteotomy group (n=16)**	**Buccal window osteotomy group** **(n=16)**	***P*** ** value**
Distobuccal	T_0_	3.53 ± 1.19	3.81 ± 1.32	0.543
	T_1_	5.23 ± 1.12	4.03 ± 1.16	0.006
Mid-distal	T_0_	4.4 ± 1.28	4.41 ± 1.4	0.98
	T_1_	5.77 ± 1.08	4.25 ± 1.35	0.002
Distolingual	T_0_	3.7 ± 1.7	3.82 ± 1.23	0.834
	T_1_	5.46 ± 1.34	3.96 ± 1.11	0.002

**Table 2:** Mean distal pocket depth difference (mm) of the second mandibular molar in each group between before and three months after surgery

**Probing** **site**	**Crestal osteotomy group (n=16)**	**Buccal window osteotomy group (n=16)**	***P*** ** value**
Distobuccal	1.7 ± 0.99	0.22 ± 0.6	< 0.001
Mid-distal	1.36 ± 0.97	-0.15 ± 0.7	< 0.000
Distolingual	1.76 ± 0.7	0.15 ± 0.43	< 0.001

## Discussion

 The surgical extraction of impacted mandibular third molars is a common surgery. Any attempt to reduce the surgical complications, including periodontal diseases, pain, trismus, swelling, and nerve damage, is essential.^10^ The present study results confirmed that the probing depth at the mandibular second molar’s distolingual, distobuccal, and mid-distal aspects was significantly less if the buccal window osteotomy was used instead of the crestal method.

 From a clinical significance viewpoint, it can be said that with the buccal window technique and extraction of the impacted tooth in a direction perpendicular to the direction in which periodontal lesions form, pocket formation can be avoided because the soft tissue around the tooth also maintains its supporting bone, which remains intact. Furthermore, it prevents food impaction and dehiscence at surgical sites, resulting in reduced inflammation and a lower risk of infection. Additionally, it reduces the likelihood of lingual nerve injury by avoiding the use of lingual flaps. Overall, this technique will be beneficial for the patient because it reduces postoperative complications and enhances periodontal health.

 Bone grafts showed promising results but imposed a high cost. Ferreira Júnior et al did not suggest inorganic bovine bone grafts to avoid periodontal lesions during this surgery because of the resorption of materials.^11^ Platelet-rich plasma demonstrated better healing and less pain, inflammation, and trismus, but the need for special equipment and blood sampling has limited its use.^12^ Collagen leads to better blood clotting, granulation tissue formation, and wound protection, but there is limited literature to support its benefits following the surgical extraction of impacted mandibular third molars.^13^ Different flap techniques have been evaluated for this purpose.^6,14,15^ The triangular flap leads to buccal tissue inflammation and edema.^7^ The SZMYD flap reduces bone loss;^16^ however, its long-term results are not hopeful.^17^ Kirtiloğlu et al^18^ reported that the SZMYD flap significantly reduces the pocket depth of the mandibular second molar, but its effect after 12 months was not significant. Baqain et al^7^ reported that both envelope and triangular flaps caused deep distal pockets, while a deeper pocket was reported after the triangular method. It should be noted that the clinical effects of any modification of the methods used during the surgical extraction of mandibular third molars should be assessed in both the long and short terms. Therefore, to compare the results of studies appropriately, the duration of follow-up should be considered.

 An osteotomy is required during the surgical extraction of the impacted third molar to access the tooth. The choice of osteotomy technique is primarily based on tooth position, impaction depth and angle, and the location of the inferior alveolar nerve. The buccal window osteotomy has been proposed to maintain the periodontal health of the second mandibular molar. Montero and Mazzaglia^4^ reported that the impaction depth is the main factor determining the change in probing depth after surgery. Other factors were suture type, tissue elevation level, and overall periodontal health of the oral cavity. It has been shown that buccal window osteotomy takes less time and leads to less pain, trismus, and swelling.^8^ A bony bridge remains in the crestal area using the buccal osteotomy technique. It reduces the apical movement of the distal pocket, even in the case of fibrous scar tissue formation or lateral movement of the epithelium towards the lesion.

 All the patients included in the present study were 18‒25 years old, with no prior periodontal disease, which indicates that they may experience faster healing after surgery due to their young age.^19^ The osteotomy technique used during surgery cannot be chosen only based on the periodontal health requirements, and this choice should be based on the surgeon’s preference, experience, and the patient’s conditions.^20^ Further studies are required to determine the benefits of this technique.

 The limitations of this study were a short-term follow-up period and reduced sample size. Our study’s three-month follow-up highlights the importance of long-term follow-ups as an essential topic for further investigation. Additionally, although the intended sample size was 26 patients, technical limitations ultimately resulted in only 16 being included. Therefore, our results were interpreted with appropriate caution.

## Conclusion

 Based on the results of this study, the probing depth at the distal aspect of mandibular second molars three months after the surgical extraction of impacted mandibular third molars using the buccal window osteotomy technique was less than that of the crystal osteotomy. Further studies on other periodontal factors, such as clinical attachment levels, plaque index, and bleeding on probing, are suggested to compare the two techniques. Studies comparing postoperative complications of the two osteotomy techniques are also recommended.

## Competing Interests

 The authors declare that they have no competing interests regarding the authorship and/or publications of this paper.

## Consent for Publication

 Not applicable.

## Data Availability Statement

 Further data are available from the corresponding author upon reasonable request via email.

## Ethical Approval

 The research was registered in the Shahid Beheshti University of Medical Sciences Ethics Committee under the code IR.SBMU.RIDS.REC.1395.410. This clinical trial was registered in the Iranian Registry of Clinical Trials (IRCT) under code IRCT20180307038996N1.
